# Toll-Like Receptor-4 Signaling Pathway Influenced the Exosome Biogenesis and Angiogenesis in Human Triple-Negative Breast Cancer Cells

**DOI:** 10.34172/apb.025.45431

**Published:** 2025-08-31

**Authors:** Parisa Khanicheragh, Çığır Biray Avci, Zahra Abbasi-Malati, Maryam Sabour Takanlou, Leila Sabour Takanlou, Reza Rahbarghazi, Akbar Hasani

**Affiliations:** ^1^Drug Applied Research Center, Tabriz University of Medical Sciences, Tabriz, Iran; ^2^Department of Clinical Biochemistry and Laboratory Medicine, Tabriz University of Medical Sciences, Tabriz, Iran; ^3^Department of Medical Biology, Faculty of Medicine, Ege University, Izmir, Turkey; ^4^Student Committee Research Center, Tabriz University of Medical Sciences, Tabriz, Iran; ^5^Stem Cell Research Center, Tabriz University of Medical Sciences, Tabriz, Iran; ^6^Department of Applied Cell Sciences, Faculty of Advanced Medical Sciences, Tabriz University of Medical Sciences, Tabriz, Iran

**Keywords:** Breast cancer, Toll-like receptor, Exosome, Angiogenesis, Migration

## Abstract

**Purpose:**

The close relationship of the toll-like receptor (TLR) signaling pathway has been indicated with different bioactivates of tumor cells. Here, the impact of TLR4 signaling pathway stimulation/inhibition was assessed on angiogenesis and exosome (Exo) biogenesis in MDA-MB-231 cells.

**Methods:**

Cells were incubated with lipopolysaccharide (LPS) and simvastatin (SIM) for 48 hours. Cell survival and TLR4 signaling pathway genes were measured using MTT and real-time PCR analysis. The physicochemical properties of Exos were studied using DLS, SEM, and western blotting. The migration capacity and angiogenesis-related genes were assessed using the Transwell insert assay and real-time PCR analysis.

**Results:**

Data indicated that SIM and LPS can reduce the survival rate in a dose-dependent manner compared to the control cells (*P*<0.05). The expression of TLR4, NF-κB, IL-1β, MYD88, and TRIF was increased in LPS-treated cells compared to the control group (*P*<0.05), while these genes were down-regulated or remained unchanged in the SIM group. SEM analysis indicated the reduction of Exo diameter in the LPS groups (*P*<0.05) with a slight increase of CD63, TSG101, and Rab27 in the presence of LPS. We found an enhanced and reduced migration rate in the LPS and SIM groups compared to the non-treated control cells (*P*<0.05). The expression of genes related to angiogenesis was down-regulated in both SIM and LPS groups.

**Conclusion:**

These data indicate that the TLR4 signaling pathway can control the angiogenesis and Exo production in breast cancer cells, which paves the way for the development of de novo therapies in breast cancer patients.

## Introduction

 Breast cancer (BC) is the second leading cause of female mortality in industrialized and developing countries.^1^ Despite the existence of several therapeutic strategies for BC patients, treatment failure increases the possibility of recurrence and metastasis to remote sites. In this regard, new therapeutic protocols with fewer side effects are mandatory.^2^ Among different BC types, triple-negative BC (TNBC) is highly aggressive with rapidly proliferating tumor cells that promote the formation of metastatic foci.^3,4^ TNBC cells lack typical membrane-bound hormone receptors such as estrogen, progesterone, and HER-2 receptors, with inherent resistance against chemotherapeutics.^5^ It is thought that these features are closely associated with the existence of distinct subsets, namely cancer stem cells (CSCs) within the tumor parenchyma. It was suggested that CSCs can stimulate tumor cell expansion and relapse several days after the administration of chemotherapeutics.^6^ Therefore, the development and selection of efficient therapeutic protocols targeting BC CSCs can circumvent BC therapeutic resistance in the clinical setting.^7^

 Based on previously published data, the bioactivity of tumor cells is under the control of several signaling pathways. Among several molecular pathways, the Toll-like receptor (TLRs) signaling pathway is involved in tumor formation and activation of resistance mechanisms.^8^ TLR signaling pathway encompasses different intracellular effectors and cell membrane-bound receptors like TLR-1, -2, -3, -4, -5, -6, -7, -8, -9, etc. Among different TLRs, TLR4 has been identified in glioma CD133^+^CSCs with the potential to stimulate the proliferation rate and evasion from CD8^+^lymphocytes.^9^ After the attachment of ligands and activation of TLR4, different cytokines and chemokines are produced inside the host cells in a MyD88- and TRIF-dependent manner.^10,11^ These effects have been shown in the acquisition of stemness features and resistance to chemotherapeutics.^12^

 Besides the existence of several intracellular mechanisms inside the CSCs to preserve the entity and development of the tumor mass, these cells can control multiple cellular processes in heterogeneous non-CSCs in a paracrine manner via the release of cytokines.^13^ Emerging data have revealed reciprocal CSC to non-CSC interaction via the release of diverse cytokines inside the extracellular vesicles (EVs).^14^ EVs include exosomes (Exos), microvesicles (MVs), and apoptotic bodies with bioactive cargos that are interchanged between the donor and recipient cells.^15^ Exos are produced by the activity of the endosomal system within the lumen of early, late endosomes, and multivesicular bodies (MVBs) via the invagination of endosome membrane and simultaneous sequestration of signaling molecules inside the intraluminal vesicles (ILVs). Following fusion with the cell membrane, ILVs are released into the surrounding niche, hereafter named Exos. These particles can easily be distributed and taken by other cells.^15^ Similar to other microenvironments, Exos can enter the tumor parenchyma and are internalized into different cells, leading to the alteration of the metabolic profile in acceptor cells.^16^ Thus, CSCs can transfer several resistance factors via Exos into the other cells, indicating the critical role of Exos in the regulation of non-CSC function.^17^

 The formation of new vascular beds inside the cancers contributes to rapid tumor cell expansion and metastasis to the neighboring tissues and remote organs.^18,19^ Exos with different molecular cargoes can control the phenomenon of neovascularization into the tumor structure.^15,20,21^ While TLR4 has been implicated in Exo production, its influence on the angiogenic potential of TNBC remains unclear. Here, in this experiment, we aimed to examine the possible stimulatory/inhibitory role of the TLR4 signaling pathway in the Exo biogenesis in human BC CSCs. Whether and how the activation/inhibition of the TLR signaling pathway can influence the Exo biogenesis and thus angiogenesis is at the center of debate. We hope that the results of this study help us to understand the possible influencing effects of TLRs on the angiogenesis behavior of BC CSCs via a paracrine manner.

## Material and methods

###  Cell culture 

 In this study, human BC MDA-MB-231 cells with typical stemness features (CD44^+^/CD24^−^) were used.^22^ Cells are purchased from the National Cell Bank of Iran (Tehran) and expanded in Dulbecco’s Modified Eagle Medium/High Glucose (DMEM/HG; Bioidea, Iran) culture medium with 10% fetal bovine serum (FBS; Biosera) and 1% Pen-Strep (Biosera) solution recommended conditions (37°C, 5% CO_2_, and 95% relative humidity). Cells were subcultured after reaching 70-80% confluence using 0.25% Trypsin-EDTA (Cat no: B11036; Bioidea). The culture medium was replaced every 3-4 days, and cells between passages 3-6 were used in different analyses.

###  Survival assay

 MDA-MB-231 cells (1 × 10^4^) were seeded in each well of 96-well culture plates and allowed to reach an appropriate confluence. To stimulate TLR4, cells were incubated with different doses of *Escherichia coli* O111: B4 strain lipopolysaccharide (LPS; Cat no: L2630 Sigma-Aldrich) and Simvastatin (SIM; Merck) for 48 hours. Cells were incubated with different doses of LPS and/or SIM in a culture medium containing 1% FBS and 1% Pen-Strep solution. After the completion of incubation time, supernatants were discarded and replaced with 200 µL MTT (5 mg/mL; Cat no: M5655; Sigma-Aldrich) and kept for 3-4 hours at 37 °C. The process was continued with the removal of the MTT solution and the addition of 100 µL of dimethyl sulfoxide solution per well. After gentle agitation, the optical density of groups was read by an automatic microplate spectrophotometer (Anthos Zenyth 340st, Austria) at 570 nm. The survival rate was expressed as % of the non-treated control group. In this study, the maximum levels of LPS (2 µg/mL) and SIM (5 mM) with less cytotoxicity were selected for subsequent analyses.

###  Exo-based analyses

####  Exo isolation 

 To this end, Exos are isolated using previously standard protocols.^23^ In brief, cells were cultured for 48 hours in Exo-free FBS. After that, the supernatants were collected and centrifuged at 300, 2000, and 10,000 g for 5, 15, and 30 minutes, respectively, to exclude cell debris and organelles, followed by passing through 0.22 µm microfilters. To yield the Exos, supernatants were centrifuged at 100,000 (Beckman Coulter Inc. Optima^TM^ TLX-120 ultracentrifuge) for 60 minutes. The exosomal pellets were collected and subjected to different assays.

####  Exo immunophenotyping using western blot analysis 

 To confirm the Exo phenotype, protein levels of CD63, CD81, and TSG101 were monitored in Exo pellets using western blot analysis. In short, the Exo samples were lysed using NP-40 lysis buffer, and protein levels were measured using the BCA method. ~10 µg of exosomal protein was electrophoresed using 10% SDS-PAGE and transferred onto the PVDF membrane. After blocking, the membranes were incubated with anti-human CD63 (Cat no: sc-5275; Santa Cruz Biotechnology Inc.), CD81 (Cat no: sc-166029; Santa Cruz Biotechnology, Inc.), and TSG101 (Cat no: sc-7964; Santa Cruz Biotechnology, Inc.) antibodies according to the manufacturer’s instructions. The targeted immunoreactive bands were visualized using ECL and X-ray films.

####  Dynamic light scattering (DLS) 

 The size and zeta potential values of isolated Exos were monitored using DLS (Model: Anton Paar Litesizer 500, Austria). The Exo stocks were diluted in a ratio of 1:5 in phosphate-buffered saline (PBS), and the desired parameters were measured.

####  Scanning electron microscopy (SEM)

 Exo samples were fixed using a 2.5% paraformaldehyde (PFA) solution, diluted in distilled water, placed on aluminum foils, and allowed to air-dry. After treatment in an ascending series of EtOH, the samples were gold-sputtered and imaged using an SEM system (Mira-3 FEG SEM microscope, Tescan Co.).

####  Exo biogenesis in BC CSCs

 In accordance with the present study objectives, MDA-MB-231 cells were collected 48 hours post-incubation with LPS and/or SIM, and total protein contents were extracted using NP-40 lysis buffer. The content of proteins in different samples was determined using the BCA assay. About 10 µg protein from each group was used for electrophoresis in a 10% SDS-PAGE gel, followed by transferring onto PVDF membranes (Bio-Rad) and blocking in 5% skim milk solution (Sigma-Aldrich) for 30-60 minutes. After that, membranes were incubated with Rab27 (Cat no: sc-74586; Santa Cruz Biotechnology Inc.), CD63 (Cat no: sc-5275; Santa Cruz Biotechnology Inc.), and ALIX (Cat no: sc-53540; Santa Cruz Biotechnology Inc.) antibodies at 4 °C overnight. After several washes with PBST, membranes were incubated with HRP-conjugated secondary antibodies to label the immunoreactive bands. To visualize the bands, X-ray films with ECL solution were used.

###  Real-time PCR analysis

 To confirm the stimulation/inhibition of the TLR4 signaling pathway in LPS- and SIM-treated MDA-MB-231 cells, the expression of genes such as TLR4, NF-κB, IL-1, MYD88, and TRIF was monitored after 48 hours. The migration properties of treated cells were also studied in terms of MMP-2 and MMP-9. Total RNA contents were extracted using TRIzol^®^ reagent (Cat no: LB38055; Life Biolab, Germany), and reverse-transcribed into cDNA using a cDNA synthetase kit (Parstous, Iran). The above-mentioned primers were designed using web-based NCBI and OligoAnalyzer^TM^ Tool (Table 1). The expression of target genes was assessed in a final reaction volume of 10 µL [5 µL SYBR Green Master Mix, forward, and reverse primers each in 0.25 µL, 1 µL sample cDNA, and 3.5 µL D.W.], and Light Cycler 480 Instrument II (Roche). The relative expression was calculated using the 2^−ΔΔCT^ formula after normalization with the GAPDH housekeeping gene.

**Table 1:** List of primers used for monitoring the TLR signaling pathway and migration capacity

**Gene**	**Forward primer (5’→3’)**	**Reverse primer (5’→3’)**	**Ref**	**Annealing (°C)**
*GAPDH*	AACATCATCCCTGCCTCTAC	CTGCTTCACCACCTTCTTG	-	60
*TLR4*	CCCTGAGGCATTTAGGCAGCTA	AGGTAGAGAGGTGGCTTAGGCT	-	60
*MyD88*	GGTGGTGGTTGTCTCTGATG	GGATGCTGGGGAACTCTTTC	-	60
*TRAF*	CAATGCCAGCGTCCCTTCCAAA	CCAAAGGACAGTTCTGGTCATGG	-	60
*NF- κ B*	GCAGCACTACTTCTTGACCACC	TCTGCTCCTGAGCATTGACGTC	-	60
*MMP9*	ACGCACGACGTCTTCCAGTA	CCACCTGGTTCAACTCACTCC	^24^	60
*MMP2*	CTCATCGCAGATGCCTGGAA	TTCAGGTAATAGGCACCCTTGAAGA	^24^	60
*IL-1*	TGTATGTGACTGCCCAAGATGAAG	AGAGGAGGTTGGTCTCACTACC	-	60

Different genes related to the TLR signaling pathway were designed in this study. The sequences for MMP-2 and MMP-9 were adapted from previously published data.

###  TLR modulation impact on Exo biogenesis and physicochemical properties 

 To this end, the intracellular levels of CD63, Alix, and Rab27 were assessed in LPS- and SIM-treated MDA-MB-231 cells using western blotting as aforementioned. The supernatant Exos were also collected from experimental groups, and different parameters were examined using SEM and DLS techniques.

###  TLR modulation and BC angiogenesis potential 

 Using PCR array analysis, the expression of AKT1, IL-8, TIMP2, CDH5, TIMP3, ERBB2, HIF1A, TNF, IFNA1, VEGFA, IFNG, NOTCH1, PECAM, and IL6 was monitored using a panel of primers according to the above-mentioned protocol (Table 2). The values of more than 2-fold changes were regarded as significant up-regulation or down-regulation.

**Table 2:** List of primers used for monitoring angiogenesis potential

**Gene**	**Primer sequencing (5'-3')**
*HPRT1*	F- GACCAGTCAACAGGGGACAT, R- GTGTCAATTATATCTTCCACAATCAAG
*B2M*	F- TAGGAGGGCTGGCAACTTAG, R- CCAAGATGTTGATGTTGGATAAGA
*GAPDH*	F- CCCCGGTTTCTATAAATTGAGC, R- CTTCCCCATGGTGTCTGAG
*ACTB*	F- AGAGCTACGAGCTGCCTGAC, R- CGTGGATGCCACAGGACT
*AKT1*	F- CCTGAACCCCATGCTCTG, R- CGGGGAGTCCAGGCTTAC
*CDH5*	F- CTTCACCCAGACCAAGTACACA, R- TGTTGGCCGTGTTATCGTGA
*HIF-1 α*	F- AGAGGTTGAGGGACGGAGAT, R- GCACCAAGCAGGTCATAGGT
*IFNG*	F- TGTAGCGGATAATGGAACTCTTTT, R- AATTTGGCTCTGCATTAT T
*PECAM-1*	F-TGAGTGGTGGGCTCAGATTG, R-TGAGTCTAGGTCGGGGAGTG
*IL-6*	F- GGTACATCCTCGACGGCATCT, R- GT GCCTCTTTGCTGCTTTCAC
*IL-8*	F- AGGGCCAAGAGAATATCCGA, R- ACTTGTGGATCCTGGCTAGC
*NOTCH1*	F- TGGACGACAACCAGAATGAG, R- TCCTCGAACCGGAACTTCT
*TGFBR1*	F- GCAGACTTAGGACTGGCAGTAAG, R- AGAACTTCAGGGGCCATGT
*VEGFA*	F- CTACCTCCACCATGCCAAGT, R- GATAGACATCCATGAACTTCACCA
*TIMP2*	F- GTGGGTCCAAGGTCCTCAT, R- CGAAGCCCCAGACACATAGT
*TIMP3*	F- CCTTCTGCAACTCCGACATC, R- GCCCCTCCTTTACCAGCTT
*ERBB2*	F- CAACTGCACCCACTCCTGT, R- GCAGAGATGATGGACGTCAG
*TNF*	F- CAGCCTCTTCTCCTTCCTGAT, R- GCCAGAGGGCTGATTAGAGA
*IFNA1*	F- AACTCCCCTGATGAATGCGG, R- AGTGTAAAGGTGCACATGACG

F: Forward sequence, and R: Reverse sequence

###  Transwell insert migration

 Treated MDA-MB-231 cells (~50,000) were resuspended in 200 µL culture medium with 1% FBS and transferred onto 8 µm Transwell inserts (SPL). In the basolateral space, 700 µL culture medium enriched with 10 ng/mL SDF-1α was poured, and cells were kept for 48 hours under standard conditions. After that, inserts were carefully removed, washed twice with PBS, and incubated in 4% PFA solution. The upper surface of the inserts was carefully cleaned using cotton swabs, and cells in the ventral surface were stained using Giemsa solution. The number of cells was counted in serial HPF, and the mean migrated cells on the ventral surface was compared among different experimental groups.

###  Statistical analysis

 In this study, data (mean ± SD) were analyzed using one-way ANOVA with Tukey post hoc test. *P* < 0.05 is considered statistically significant. 

## Results

###  LPS and SIM reduced MDA-MB-231 cell viability in a dose-dependent manner

**Figure 1:**
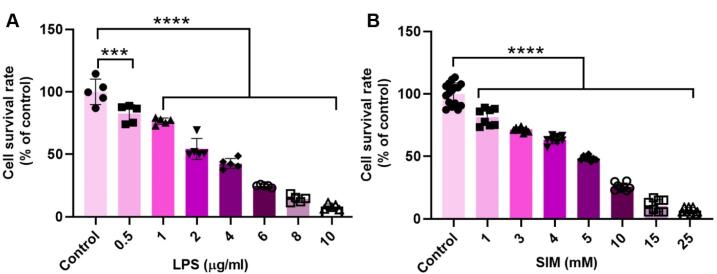
MTT assay. The viability of human MDA-MB-231 cells was monitored using MTT after being exposed to different doses of LPS (**A**; n = 5) and SIM (**B**; Replicate for the control cells and other groups is 16 and 8, respectively) for 48 hours. One-way ANOVA with Tukey post hoc analysis. ***P < 0.001; and ****P < 0.0001

 In this study, an MTT assay was used to assess the toxic effects of LPS and SIM on human MDA-MB-231 cells (Figure 1A-B). In this regard, MDA-MB-231 cells were exposed to different doses of LPS (0.5 to 20 μg/mL) and SIM (1 to 25 mM) for 48 hours. Data showed a significant reduction of MDA-MB-231 viability in a dose-dependent manner after being exposed to LPS and SIM compared to the control group (*P* < 0.05). MDA-MB-231 cells treated with 10 μg/mL LPS showed less survivability compared to the other groups. Treatment of MDA-MB-231 cells with 0.5 μg/mL LPS had slight toxic effects as compared to the increasing doses of LPS. A similar pattern was shown in this line upon treatment of MDA-MB-231 cells with SIM, in which the 25 mM SIM group had greater than 95% of cells dead after 48 hours. These data indicate that both SIM and LPS can exert tumoricidal properties on human BC cells in a dose-dependent manner. According to data from current experiments and previous studies, 2 μg/mL LPS and 5 mM SIM were used for activation and inhibition of TLR4 signaling pathways in different analyses.

###  Exo characterization and immunophenotyping 

**Figure 2:**
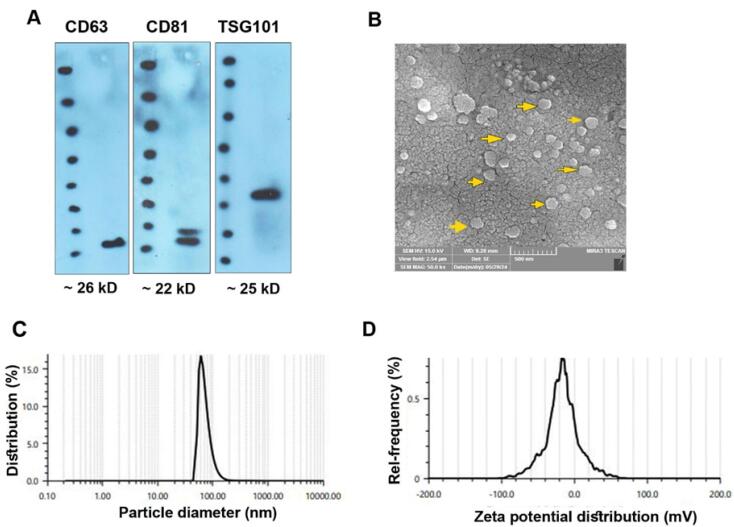
Characterization of MDA-MB-231 Exos using western blotting (**A**), SEM (**B**), and DLS (**C** and **D**). Western blotting indicated typical Exo markers CD63, CD81, and TSG101 (**A**). SEM images revealed the heterogeneous size of isolated Exos (**B**; yellow arrows). Using DLS, a mean diameter size of 79.9 ± 27.57 nm, and a zeta potential value of -16.7 mV were achieved

 Using different analyses, the physicochemical properties of isolated Exos from MDA-MB-231 cells were monitored (Figure 2A-D). Based on the western blotting panel, the MDA-MB-231 cell Exos were positive for tetraspanins CD63, CD81, and TSG101, indicating the validity of the present protocol in the isolation and enrichment of Exos from human BC cells (Figure 2A). SEM images revealed that isolated human MDA-MB-231 cell Exos exhibit round spheroid morphology with heterogeneity in size (Figure 2B; yellow arrows). Based on our calculation, the mean Exo diameter was 58.63 ± 27.57 nm (Figure 2B). DLS revealed that the hydrodynamic diameter of isolated Exos reached 79.9 ± 27.57 nm with zeta potential values of -16.7 mV (Figure 2C-D). These data demonstrated the typical physicochemical properties related to isolated Exos from human MDA-MB-231 cells.

###  TLR4 signaling pathway activation and inhibition 

####  LPS can stimulate the expression of TLR4 signaling pathway genes

**Figure 3:**
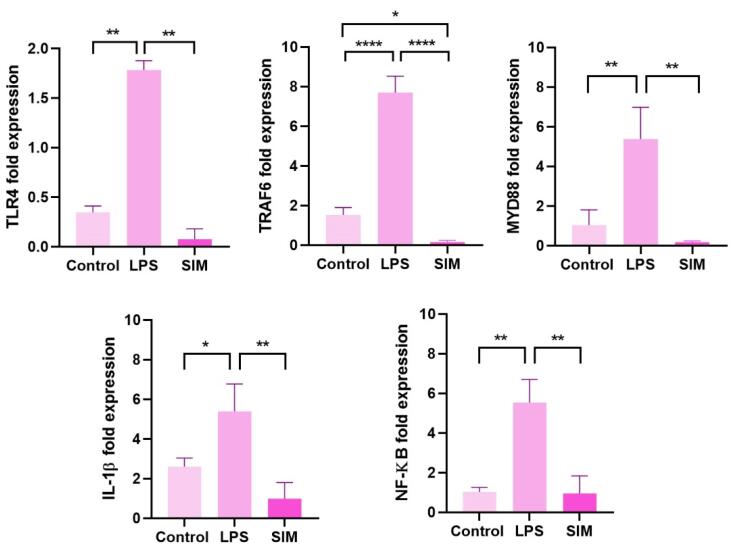
The expression of TLR signaling pathway effectors was measured using real-time PCR analysis. One-way ANOVA with Tukey post hoc analysis (n = 3). * P < 0.05; ** P < 0.01; and **** P < 0.0001

 To confirm whether the selected doses of LPS (2 μg/mL) and SIM (5 mM) can influence the expression of TLR4, TRAF6, MYD88, IL-1β, and NF-ĸB belonging to the TLR4 signaling pathway, real-time PCR analysis was done after 48 hours (Figure 3). Data revealed that 48-hour incubation of human MDA-MB-231 cells with 2 μg/mL LPS led to the activation of the TLR4 signaling pathway in which the expression of TLR4, MYD88, NF-κB, IL-1β, and TRAF6 was statistically increased compared to the non-treated control cells (*P* < 0.05). It was also noted that 5 μM SIM led to a non-significant and slight reduction in the expression of TLR4, TRAF6, MYD88, IL-1β, and NF-ĸB when compared to the control group (*P* > 0.05) (Figure 3). These data indicated that LPS and SIM had the potential to stimulate and inhibit the TLR signaling pathway, respectively. The stimulatory effects of LPS in the TLR4 signaling pathway are mediated by the up-regulation of TLR4, TRAF6, MYD88, IL-1β, and NF-ĸB.

####  TLR4 signaling pathway stimulation increased Exo biogenesis in MDA-MB-231 cells

**Figure 4:**
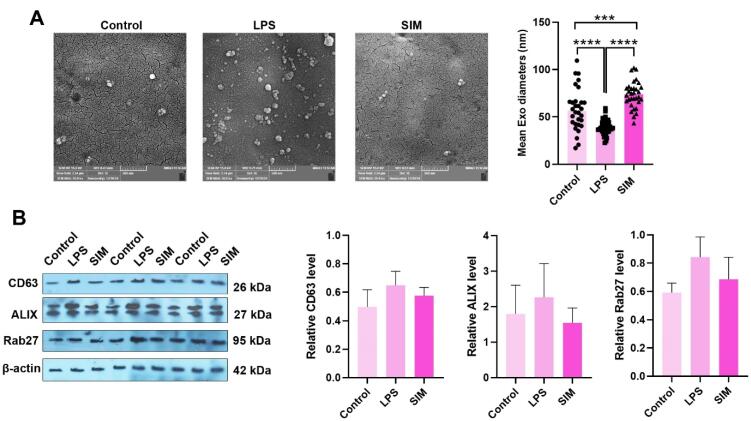
Exos were imaged using SEM in MDA-MB-231 cells after being treated with LPS and SIM for 48 hours (**A**). The number of Exo was increased per field of SEM images in the LPS groups compared to the control and SIM groups. Data indicated the reduction and increase of mean Exo diameter in LPS and SIM groups, respectively (Control: n = 30; LPS: n = 37; and SIM: n = 33). Western blotting (**B**). Protein levels of CD63, ALIX, and Rab27 were measured in MDA-MB-231 cells 48 hours after treatment with LPS and SIM. Data indicated a slight but non-significant increase in the target protein in the LPS group. (n = 3). One-way ANOVA with Tukey post hoc analysis (n = 3). *** P < 0.001; and **** P < 0.0001

 The possible stimulation/inhibition effects of the TLR4 signaling pathway were monitored in Exo biogenesis in MDA-MB-231 cells (Figure 4A-B). SEM images indicated the changes in Exo number per field in LPS-treated MDA-MB-231 cells compared to the control and SIM groups (Figure 4A). Based on the data, the number and heterogeneity of Exos increased in the LPS-treated MDA-MB-231 cells, in which both small- and large-sized Exos could be identified in captured images. In contrast to the LPS group, the number of isolated Exos was less in SIM-treated and control supernatants. In line with these changes, the Exo population heterogeneity was also reduced in these groups in comparison with the LPS-treated cells. Based on the data, treatment of MDA-MB-231 cells with 2 μg/mL LPS statistically reduced the mean Exo diameter compared to other groups (*P* < 0.05). In contrast, the incubation of cells with 5 μM SIM increased the mean Exo size compared to the control and LPS-treated MDA-MB-231 cells after 48 hours. These data indicate that the stimulation and inhibition of TLR4 can influence the mean Exo size released by human MDA-MB-231 cells. Western blotting of parent MDA-MB-231 cells showed a non-significant slight increase in protein levels of CD63, ALIX, and Rab27 in the LPS groups (Figure 4B). Meanwhile, these changes were similar in the SIM-treated cells as compared to the control group. These data show that the stimulation and inhibition of the TLR4 signaling pathway can alter the number and size of Exos without significant changes in the protein levels of tetraspanins in the donor BC cells.

###  TLR4 signaling pathway stimulation promoted BC cells’ migration 

**Figure 5:**
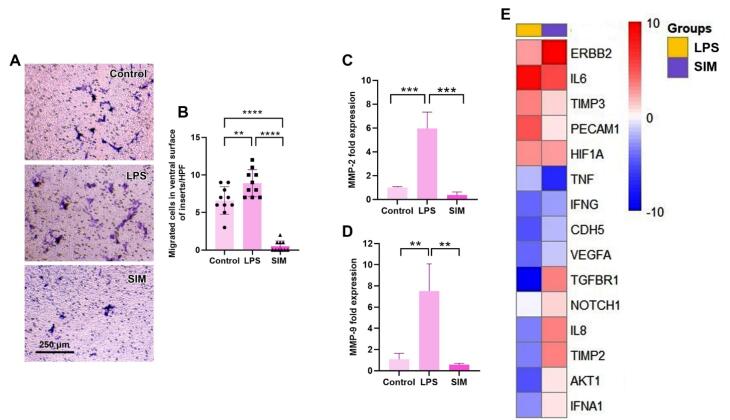
Transwell insert assay (**A** and **B**). Monitoring the number of migrated MDA-MB-231 cells on the ventral surface of inserts after 48 hours (n = 10). The expression of MMP-2 and MMP-9 in MDA-MB-231 cells after treatment with LPS and SIM for 48 hours (**C** and **D**; *n* = 3). One-way ANOVA with Tukey post hoc analysis (*n* = 3). ** P < 0.01; *** P < 0.001; and ****P< 0.0001. Clustergram mapping of angiogenesis-related genes in MDA-MB-231 cells treated with SIM and LPS for 48 hours (**E**; *n* = 3)

 To assess whether treatment of MDA-MB-231 cells with LPS and/or SIM for 48 hours can influence migration capacity, the number of migrated cells was counted in Transwell inserts in the presence of SDF-1α (Figure 5A). Data showed enhanced migration properties in MDA-MB-231 cells after treatment with LPS compared to the control and SIM-treated cells (*P* < 0.05). Unlike LPS groups, the number of migrated MDA-MB-231 cells into the ventral surface of Transwell inserts was significantly reduced as compared with the LPS and control groups (*P* < 0.05). These features show that LPS and SIM can increase and reduce the migration of human MDA-MB-231 cells, respectively, in *in vitro* systems. Besides the Transwell insert assay, the expression of MMP-2 and MMP-9 was also monitored using real-time PCR analysis (Figure 5C and D). Similar to the Transwell insert assay, the expression of MMP-2 and MMP-9 was significantly up-regulated in the presence of LPS compared to the non-treated cells (*P* < 0.05), while non-significant differences were obtained in the expression of these genes between SIM and control groups (*P* > 0.05). These data indicate that the activation of the TLR4 signaling pathway via LPS can increase the migration of BC cells via the expression of MMP-2 and MMP-9.

###  TLR4 inhibition and stimulation can reduce angiogenesis properties 

 The angiogenesis potential of MDA-MB-231 cells was also assessed by monitoring the expression of different pro-angiogenesis and anti-angiogenesis genes (Table 3 and Figure 5E). Data indicated global down-regulation of pro-angiogenesis and anti-angiogenesis genes in MDA-MB-231 cells after being exposed to LPS and SIM for 48 hours. Which incubation of MDA-MB-231 cells with 2 µg/mL LPS led to a reduction of AKT1, IL-8, CDH5, IFNA1, VEGFA, and IFNG, while these features coincided with concomitant suppression of anti-angiogenesis genes such as TIMP2 and TGFBR1. Among the genes, IL-6 was significantly upregulated compared to the control MDA-MB-231 cells, indicating the inflammatory response. In the SIM-treated cells, the expression of most genes remained unchanged, except that TNF-α and IFNG were significantly down-regulated. Inexpertly, the expression of pro-angiogenesis factor ERBB2 was stimulated compared to the control group, while these changes were statistically nonsignificant in the LPS group. These data indicate that the stimulation/inhibition of the TLR signaling pathway for 48 hours can impair the angiogenesis potential in human BC MDA-MB-231 cells after 48 hours *in vitro*.

**Table 3:** Expression of pro- and anti-angiogenesis-related genes in MDA-MB-231 cells 48 hours after treatment with SIM and/or LPS

**Gene**	**Fold changes compared to the control MDA-MB-231 cells**	
	**LPS**	**SIM**
*AKT1*	**-3.06**	-0.89
*IL8*	**-2.56**	0.21
*TIMP2*	**-2.47**	0.20
*CDH5*	**-3.18**	-1.89
*TIMP3*	0.21	-0.81
*ERBB2*	-0.14	**2.72**
*HIF1A*	0.03	-0.14
*TNF*	-1.84	**-3.84**
*IFNA1*	**-2.40**	-0.89
*VEGFA*	**-2.74**	-1.74
*IFNG*	**-2.74**	**-2.25**
*ACTB*	0.00	0.00
*NOTCH1*	-1.29	-0.84
*B2M*	2.41	-2.47
*PECAM1*	0.72	-0.89
*GAPDH*	4.11	4.72
*IL6*	**2.26**	0.96
*TGFBR1*	**-5.06**	0.08
*HPRT1*	6.87	6.31

Bold numbers: The significant upregulation and downregulation

## Discussion

 It has been thought that the TLR signaling pathway, with several intracellular and cell-membrane receptors with pleiotropic effects, has overlapping and crosstalk with other molecular cascades.^25^ Here, we aimed to address possible synergetic or inhibitory interplay between the TLR signaling pathway, with Exo biogenesis, and angiogenesis behavior in human MDA-MB-231 cells *in vitro*. For this purpose, SIM and LPS were used as TLR4 signaling pathway inhibitors,^26^ and stimulators,^27^ respectively. Our data indicate that the increasing doses of LPS led to a reduced survival rate in MDA-MB-231 cells after 48 hours. Due to the intricacy and complexity of the TLR signaling pathway, a multitude of cellular effects, such as tumoricidal effects and tumor cell protection, have been reported after stimulation of TLR4.^28^ Previous data have indicated different mechanisms involved in the tumoricidal properties of LPS via TLR4 signaling pathways. For instance, LPS can promote apoptotic changes via the reduction of inhibitors of apoptosis (IAP) and TNF-α production, in which these effects are intensified in the presence of IAP antagonists.^29^ In monocytic THP-1 cells exposed to 1 µg/mL LPS for 24 hours, the activation of intrinsic (caspase 9) and extrinsic (caspase 8) apoptotic pathways contributed to cell death via the TLR4/CD14-dependent signaling axis.^30^ Domenis et al found that treatment of MDA-MB-231 cells with 1 µg/mL LPS for 24 hours led to a 25% viability reduction.^31^ Conversely, Haricharan and co-workers declared that the activation of TLR4 with LPS in p53 mutant BC MDA-MB-231 cells can lead to the proliferation of these cells via the production of GM-CSF and expression of S100A7.^32-34^ One reason for these opposite results would be that distinct subpopulations have been identified in TNBC cell lines such as MDA-MB-231 cells with different tumorigenicity, resistance (CD44^+^/CD24^-^ versus CD44^+^/CD24^low^) to insulting conditions. Therefore, we hypothesize that the CD44^+^/CD24^-^ phenotype is more sensitive to the induction of TLR4 compared to CD44^+^/CD24^-^ cells.^35^ It is also possible that the opposite difference in survival rates can be related to the incubation time and various doses of LPS used in the present study compared to the previous experiments. Therefore, future studies should focus on the time and dose-dependent activity of LPS and TLR4 signaling pathways on the dynamic growth of the BC cells. Along with these data, incubation of MDA-MB-231 cells with 5 mM SIM reduces the survival rate in a dose-dependent manner. Based on the previous data, SIM can blunt the glycolysis potential in MDA-MB-231 cells, which exhibit higher glycolytic activity compared to the other BC cell lines, like MCF-7 produce their energy via mitochondrial activity.^36^ The changes in lipid content and synthesis are related to the reduced viability in cancer cells after treatment with SIM.^37^ The stimulation of purinergic receptor P2X7/Akt signaling axis is another tumoricidal property of the statin family.^38^

 Real-time PCR analysis revealed the activation of the TLR4 signaling pathway in the presence of LPS via the activation of the MyD88-dependent pathway and up-regulation of genes such as TLR4, TRAF6, MYD88, IL-1β, and NK-ĸB. It has been shown that the direct physical interaction of LPS with adaptor protein, namely myeloid differentiation factor-2 (MD-2), accelerates the formation of LPS/TLR4/MD-2, resulting in triggering downstream signaling cascades.^27^ Even though different studies demonstrated that exposure of MDA-MB-231 cells to LPS can contribute to the up-regulation of TLR4, TLR4 is highly expressed in these cells compared to other BC cell lines.^28,31^ On the contrary, compared to the LPS groups, the expression of all monitored genes in this study was significantly reduced, indicating the inhibition of the TLR4 signaling pathway. Jarrett and co-workers found that the inhibition of the TLR4/NF-ĸB signaling axis in the presence of SIM can result in diminished osteogenic response in aortic valve interstitial cells.^39^ Data from the present experiment and previous studies indicate that statins such as SIM can efficiently blunt the activity of the TLR4 signaling pathway.

 The dynamic interplay between Exo biogenesis and the TLR4 signaling pathway was also studied in the present study. SEM analysis revealed the reduction of mean Exo diameter after the stimulation of TLR4 compared to the control and SIM groups. Besides, the number of Exos was also diminished in the same dilutions prepared from different experimental groups. Along with these changes, a slight increase but no significant changes were evident in LPS groups when compared to the control and SIM groups. Previous data indicated that activation of MDA-MB-231 cells with µg/mL LPS for 25 hours did not influence the number of Exos released.^31^ It seems that the slight increase in the expression of Exo biogenesis factors such as CD62, Alix, and Rab27 stands for the fact that Exo production and release are initiated in the presence of LPS. In support of this notion, it was suggested that treatment of mesenchymal stem cells with 100 ng/mL LPS can lead to an increase in Exo production with an increase of exosomal protein content of ~37%.^40^ It is believed that the IRF-1 factor is stimulated along with the activation of the TLR signaling pathway.^41^ It has been shown that IRF-1 can, *per se,* promote the synthesis of Rab27a in hypoxic hepatocytes, leading to enhanced EV release.^42^ About 2.26-fold of IL-6 expression was found in the presence of LPS for 48 hours. This cytokine was shown to increase the release of Exos from primary cultured macrophages pre-treated with palmitic acid.^43^ Because the induction of the TLR signaling pathway can mimic the proinflammatory conditions caused by LPS, it can be hypothesized that these conditions can lead to an increase in exosome biogenesis in the host cells.^44^ It is assumed that the significant reduction of mean Exo diameter can be related to accelerated Exo biogenesis and reduction of transit time inside the MDA-MB-231 cells in the presence of LPS. However, future studies should address any close possible correlation between the TLR signaling pathway induction and any changes in the physicochemical properties of Exos.

 Further analyses revealed the stimulation of migration capacity in MDA-MB-231 cells (MMP-2↑ and MMP-9↑) compared to the control and SIM-treated MDA-MB-231 cells. It has been indicated that the activation of the TLR4/STAT3 signaling axis can increase migration capacity in hepatocarcinoma cells.^45^ Tripathi and co-workers found that the metastatic properties of MDA-MB-231 cells were also decreased in the presence of 20 µM SIM.^36^ The reduced prenylation of Ras pathways coincided with the inhibition of CDK4/6 and Cyclin D1 is evident after treatment with SIM, resulting in the control of tumor cell migration.^36^ Despite the increase in migration and metastatic behavior of LPS-treated MDA-MB-231 cells, the expression of several angiogenesis-related genes was down-regulated in both LPS and SIM groups. While the expression of IL-6 and ERBB2 increased in the LPS and SIM groups, respectively. Recent data confirmed that the stimulation of the TLR4 signaling pathway can contribute to inflammatory angiogenesis response in different cells.^46^ The activity of NF-κB is integral to angiogenesis properties in the host cells.^47^ As a common belief, the blockade of NF-κB coincides with the reduction of angiogenesis in different cells, especially tumor cells.^48^ However, in the present study, this activation did not influence the expression of HIF-1α and VEGF as early-stage angiogenesis cytokines. It seems that in this study, cells were exposed to higher tumoricidal doses of LPS, which can contribute to the overactivation of NF-ĸB, which could not stimulate the angiogenesis genes, but also suppress the most studied genes. In support of this notion, Tabruyn et al claimed that the activation of this factor is critical for the inhibition of angiogenesis in the host cells when co-incubated with angiostatic agents such as endostatin, anginex, angiostatin, prolactin, etc.^49^ Therefore, it can be said that the increase of IL-6, common angiogenesis, and inflammatory response is directly associated with the TLR4 signaling pathway.

 The current study faces some limitations that need further investigation to address the issues. The current isolation method may raise the possibility of protein aggregates or non-exosomal vesicles in the isolated samples. Thus, it is suggested that future studies use novel technologies to reduce impurities. Besides, further studies must explore the existence of different pro- and anti-angiogenesis factors inside the Exos released from tumor cells after stimulation and inhibition of the TLR signaling pathway. Due to the existence of several intracellular and transmembrane receptors belonging to the TLR signaling pathway, the exact pro- and anti-angiogenesis properties should be addressed under physiological and pathological conditions. The contradictory data on migration status and angiogenesis capacity of MDA-MB-231 cells treated with LPS should be answered the further studies. Whether the TLR4 signaling pathway has a greater impact on the angiocrine capacity of MDA-MB-231 cells compared to metastatic behavior should be elucidated.

## Conclusion

 In summary, the present data confirmed that TLR4 activation/inhibition can influence the Exo biogenesis, migration, survival rate, and angiogenesis potential of TNBC MDA-MB-231 cells *in vitro*. These data possibly support the importance of this signaling pathway in BC development and propagation to remote sites. Considering the present, enormous previous data, it seems that TLR4 can exert dual effects on BC cells in terms of angiogenesis. Based on the incubation time and doses of TLR signaling pathway inhibitors/stimulators, tumor cell bioactivities can be different. Taken together, precise and sophisticated regulation of TLR signaling pathways using different pharmaceuticals and chemicals could be at least a strategic plan for the control of BC development and expansion, along with conventional medications and therapeutic regimes.

## Competing Interests

 The authors declare that they have no known competing financial interests or personal relationships that could have appeared to influence the work reported in this paper.

## Consent for Publication

 Not applicable.

## Ethical Approval

 All phases of this study were approved by the ethical code of the IR.TBZMED.VCR.REC.1401.402 from Tabriz University of Medical Sciences and Vice President, Scientific Technology and Knowledge Council for Development of Regenerative Medicine and Stem Cells Technologies.
